# Effects of Elevated *p*CO_2_ on the Survival and Growth of *Portunus trituberculatus*

**DOI:** 10.3389/fphys.2020.00750

**Published:** 2020-07-10

**Authors:** Weichuan Lin, Zhiming Ren, Changkao Mu, Yangfang Ye, Chunlin Wang

**Affiliations:** ^1^Key Laboratory of Applied Marine Biotechnology, Ningbo University, Chinese Ministry of Education, Ningbo, China; ^2^Collaborative Innovation Center for Zhejiang Marine High-Efficiency and Healthy Aquaculture, Ningbo, China

**Keywords:** metabolomics, microbiota, nuclear magnetic resonance (NMR), ocean acidification, swimming crab

## Abstract

Identifying the response of *Portunus trituberculatus* to ocean acidification (OA) is critical to understanding the future development of this commercially important Chinese crab species. Recent studies have reported negative effects of OA on crustaceans. Here, we subjected swimming crabs to projected oceanic CO_2_ levels (current: 380 μatm; 2100: 750 μatm; 2200: 1500 μatm) for 4 weeks and analyzed the effects on survival, growth, digestion, antioxidant capacity, immune function, tissue metabolites, and gut bacteria of the crabs and on seawater bacteria. We integrated these findings to construct a structural equation model to evaluate the contribution of these variables to the survival and growth of swimming crabs. Reduced crab growth shown under OA is significantly correlated with changes in gut, muscle, and hepatopancreas metabolites whereas enhanced crab survival is significantly associated with changes in the carbonate system, seawater and gut bacteria, and activities of antioxidative and digestive enzymes. In addition, seawater bacteria appear to play a central role in the digestion, stress response, immune response, and metabolism of swimming crabs and their gut bacteria. We predict that if anthropogenic CO_2_ emissions continue to rise, future OA could lead to severe alterations in antioxidative, immune, and metabolic functions and gut bacterial community composition in the swimming crabs through direct oxidative stress and/or indirect seawater bacterial roles. These effects appear to mediate improved survival, but at the cost of growth of the swimming crabs.

## Introduction

Crustaceans face a range of variable environmental stressors during their complex life cycle. Temperature and salinity are commonly considered as the most important abiotic factors for the survival, growth, and reproduction of crustaceans (Ye et al., 2014; Prymaczok et al., 2016). However, ongoing ocean acidification (OA) may entail a new challenge for them. According to Sabine et al. (2004), the OA is a consequence of the absorption by oceans of atmospheric carbon dioxide (CO_2_) due to anthropogenic activities such as cement production and fossil fuels utilization. A substantial rise in oceanic CO_2_ partial pressure (*p*CO_2_) has already led to a reduction of 0.1 units in the current pH of surface seawater compared to preindustrial levels. It is predicted that by 2100, there will be a further decrease of 0.3–0.5 pH units (Caldeira and Wickett, 2003; Orr et al., 2005).

The impacts of OA are raising increasing concerns with regard to crustaceans because of the sensitivity of calcifying animals (Hendriks et al., 2010). Among them, crabs present species-specific responses to OA, such as different impacts on calcification (Walther et al., 2010; Long et al., 2013a; Wei et al., 2015), and survival was shown to be reduced in crabs following a longer term exposure (months) to OA, although shorter term exposure (less than 1 month) did not have any apparent effects (Ceballos-Osuna et al., 2013; Long et al., 2013a; Ragagnin et al., 2018; Ramaglia et al., 2018). In addition, OA can also induce negative effects on crab fertilization, embryonic development, and behavior (de la Haye et al., 2011, 2012; Ceballos-Osuna et al., 2013; Long et al., 2013b; Dodd-Luke et al., 2015).

In general, such morphological and behavioral changes in crabs are associated with physiological changes. When exposed to OA, crabs sense the decreasing extracellular pH in tissues caused by OA (Wheatly and Henry, 1992; Pane and Barry, 2007). There is evidence that efficient acid–base, metabolic, and ionic regulation contributes to the compensation of extracellular pH changes, as shown in the velvet swimming crab, *Necora puber* (Spicer et al., 2007; Small et al., 2010), the dungeness crab, *Metacarcinus magister* (Hans et al., 2014), and the shore crab, *Carcinus maenas* (Maus et al., 2018). However, such osmoregulatory changes result in a rising energy cost and the reallocation of energy thereby compromises growth and behavior (Caldeira and Wickett, 2003; Dodd-Luke et al., 2015; Ragagnin et al., 2018). Extracellular anisosmotic regulation and intracellular isosmotic regulation have been observed in crustaceans in response to environmental osmolality changes (Ragagnin et al., 2018). However, OA has a depressing effect on oxygen consumption, which subsequently affects ATP production (Pörtner et al., 2004; Carter et al., 2013; Maus et al., 2018). The activity of energy-dependent osmoregulating enzymes such as Na^+^/K^+^-ATPase and V-type ATPase therefore decreases under acidified conditions (Whiteley et al., 2001; Hu et al., 2016). In this case, free amino acids are probably used as osmotic effectors. In addition, lactate has been shown to vary in spider crabs, *Hyas araneus*, exposed to elevated *p*CO_2_ (Zittier et al., 2013). Overall, the underlying metabolic effects of OA have not been thoroughly evaluated in crabs.

The swimming crab, *Portunus trituberculatus* (Crustacea, Decapoda, Brachyura), is a widely cultured and consumed species in China with a yield of 617,540 tons in 2017 (China Fishery Statistical Yearbook, 2018). Our previous studies have shown that elevated *p*CO_2_ (750 and 1500 μatm) has significant effects on the carapace of juvenile swimming crabs (e.g., a simplified arrangement of spinules, a reduced thickness, and an increased chitin content) (Ren et al., 2017) and their behavior (e.g., an increase in shoal average speed, a preference for dark environments and fast exploration) (Ren et al., 2018). Furthermore, previous studies have reported that OA can induce oxidative stress (Wang et al., 2009; Menu-Courey et al., 2019) and suppress immunity (Hernroth et al., 2012) in other crustaceans. Therefore, a holistic study is needed to advance our understanding of how OA exposure affects the swimming crab.

In this study, we subjected swimming crabs to increasing CO_2_ levels for 4 weeks to simulate OA. Our aim was to extensively explore the effects of OA exposure on the survival, growth, digestion, antioxidant capacity, immune function, tissue metabolites, and gut bacteria of swimming crabs as well as on seawater bacteria using biochemical assays, real-time quantitative polymerase chain reaction (RT-qPCR), 16S rRNA gene sequencing, and nuclear magnetic resonance (NMR)-based metabolomics analysis. This study provides new evidence that OA has a positive effect on the survival but a negative effect on the growth of swimming crabs through changes in crab digestion, antioxidant capacity, immune function, gut bacteria, and metabolites and the modulation of the seawater bacteria.

## Materials and Methods

### Crabs and OA Exposure

Four hundred and twenty male juvenile stage VII swimming crabs (93.3 ± 10.0 mm carapace length [CL]) were purchased from a local aquaculture farm in Fenghua, Ningbo in Eastern China in late July 2018. Each crab was kept in a plastic box (210 × 170 × 80 mm) with small holes. These crabs were cultured in three tanks (1.5 × 1.2 × 0.6 m) containing 500 L of aerated and filtered local seawater per tank at ambient salinity (27.0 ± 0.4%) for a 1-week acclimatization. To avoid heat stress to the crabs, a chiller (Guangli Cooling Equipment Company, Guangzhou, China) was used to maintain the seawater temperature at 28°C via the heat exchange between seawater and tap water. The crabs were fed with fresh clams (*Ruditapes philippinarum*) once daily between 5:00 and 6:00 p.m., and waste food was removed before feeding.

**FIGURE 1:**
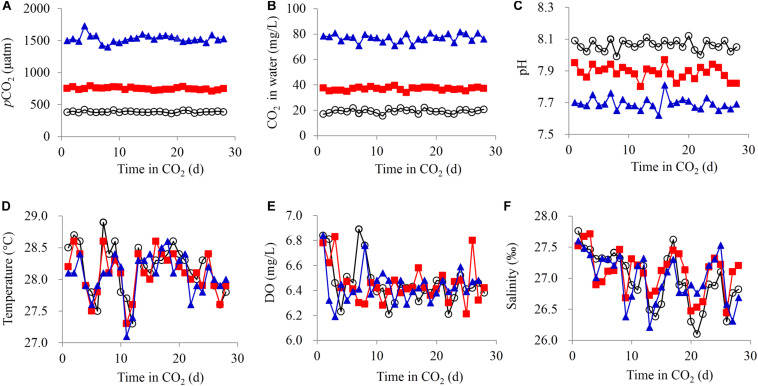
Water parameters of the carbonate system. **(A)** *p*CO_2_, **(B)** free CO_2_, **(C)** pH, **(D)** temperature, **(E)** dissolved oxygen (DO), and **(F)** salinity.

Three experimental OA groups were designed with seawater *p*CO_2_ at current or predicted future levels: the ambient *p*CO_2_ was 380 μatm (designated the 380 group), 750 μatm (designated the 750 group) was used to represent the scenario at the end of this century and 1500 μatm (designated the 1500 group) was used to represent the scenario in c. 2200 (Revi et al., 2014; Gattuso et al., 2015). To build the experimental systems, a simulated ocean acidification system (CN: ZL201520071087.1) (Ren et al., 2018) and an incubation system that was partially derived from the experimental system of Wang et al. (2018) were used. For each group, the incubation system per group consisted of a mixing tank (500 L) and three individual culture tanks (300 L), and seawater was recirculated and purified by using a water purification header tank connected to the culture tanks (200 L). The CO_2_ gas was first mixed with air in the mixing tank using a gas flow adjustment system (AK Biotechnology Co., Ltd., Qingdao, Shandong, China), followed by bubbling into seawater via a nano-aeration pipe. Both the mixing tank and the header tank were continuously bubbled with the respective and stable air–CO_2_ mixture. Each incubation system was stocked with 130 crabs with strong vitality and intact limbs with 43 crabs for two of the culture tanks and 44 crabs for the third culture tank. The flowing CO_2_ was adjusted to maintain the designed *p*CO_2_ level, and this gradually changed the pH from to the required level within 12 h. Three seawater inputs with different *p*CO_2_ levels for the three incubation systems were obtained: 380 μatm (pH = 8.1) for the control group, 750 μatm (pH = 7.9), and 1500 μatm (pH = 7.6) (Figures 1A,B). The *p*CO_2_ levels were read daily from the gas flow adjustment system. Measurements of free CO_2_ concentrations in the seawater were determined daily by a base titration method following the protocol outlined in the Water Quality Analytical Methods (SL80-1994). The swimming crabs were checked daily for death or molting. The dead crabs and exuviae were removed from the tanks. Partial water exchange (25%) was performed every 2 days in each culture tank.

### Water Quality, Sample Collection, Growth Performance, and Survival Rate

Water temperature, dissolved oxygen (DO), pH, and salinity were measured daily by using a handheld multiparameter water quality analyzer (YSI, Yellow Springs, OH, United States). The planktonic microbial biomass in the water was collected once a week during the experiment. For each group, approximately 500 mL of water sampled from the three culture tanks was filtered through a sterilized 100-μm pore nylon net, followed by a 0.22-μm pore polycarbonate filter (MilliporeSigma, Burlington, MA, United States). The filters were stored in sterilized tubes, immediately snap-frozen in liquid nitrogen, and stored at −80°C until further analysis. For each group, 20 crabs were randomly collected (6–7 crabs from each culture tank) at each sampling point (1, 2, 3, and 4 weeks) and sacrificed by being placed on ice. The CL, carapace width (CW), and body height (BH) were measured at the last sampling point (4 weeks). Their individual weights were determined for weight gain. Mortality was recorded during the experiment. Gut, muscle, and hepatopancreas tissues were collected from each sampled crab, immediately snap-frozen in liquid nitrogen, and stored at −80°C until further analysis. The gut samples were used for metabolite analysis (*n* = 6) and for bacterial analysis (*n* = 6). The muscle samples were used for metabolite analysis (*n* = 6). The hepatopancreas samples were used for metabolite analysis (*n* = 6) and enzymatic and gene expression analysis (*n* = 6).

In this study, all the procedures complied with Chinese law pertaining to experimental animals. The protocol was approved by the Ethics-Scientific Committee for Experiments on Animals of Ningbo University.

### Biochemical Assays

Hepatopancreas samples were homogenized using a TGrinder (TIANGEN, Beijing, China) in 5-mL centrifuge tubes with 9 volumes (v/w) of 0.9% physiological saline. The homogenates were centrifuged at 3750 rpm and at 4°C for 10 min. The resultant supernatants were collected and subsequently frozen at −80°C for biochemical assays. Trypsin, lipase, amylase, superoxide dismutase (SOD), glutathione-*S*-transferase (GST), malondialdehyde (MDA), total antioxidant capacity (T-AOC), acid phosphatase (ACP), and alkaline phosphatase (AKP) were analyzed using specific commercial assay kits (Nanjing Jiancheng Institute, Nanjing, Jiangsu, China).

### mRNA Expression Analysis of Oxidative, Stress, and Immunity Genes

Total RNA from hepatopancreas samples was extracted using TRIzol Reagent (Invitrogen, Waltham, MA, United States) according to the manufacturer’s protocol. The RNA quality and quantity were assessed using a NanoDrop 2000 Spectrophotometer (Thermo Fisher Scientific, Waltham, MA, United States) and the OD_260__/__280_ values of all the RNA samples ranged from 1.86 to 2.02. The isolated RNA was then treated with RQ1 RNase-free DNase (Promega, Durham, NC, United States) to eliminate DNA contamination. First-strand cDNA synthesis in reverse transcription was performed using an M-MLV reverse transcriptase (Promega, Durham, NC, United States) according to the manufacturer’s instructions. RT-qPCR was performed on a Roche LightCycler 480 Real-time PCR System (Roche, Basel, Switzerland) using a Promega GoTaq^®^ qPCR Master Mix (Promega, Durham, NC, United States). One microliter of the resultant dilution was added as a template in a final volume of 25 μL. The reactions were carried out on a quantitative thermal cycler using SYBR green I as a fluorescent dye. The PCR conditions were as follows: 95°C for 5 min for DNA denaturation, with the amplification lasting 40 cycles (95°C for 15 s, 60°C for 15 s, and 72°C for 15 s), and final extension for 10 min at 72°C. The specificities of the PCR products were detected by melting curve analysis and sequencing. In this study, β-actin was used as a reference gene. To ensure the specificity of the intended genes, all the primers for PCR were designed to span an intron. All the primer sequences used for the PCR analyses of the genes were synthesized by the Beijing Genomics Institute (Shanghai, China) and are listed in Supplementary Table S1. Each PCR run was performed in triplicate and reactions without cDNA were included as negative controls. The values obtained from PCR were analyzed using the 2^–Δ^ ^Δ^ ^*CT*^ method (Livak and Schmittgen, 2002).

### Gut and Seawater Microbiota Analysis

The bacterial genomic DNA in the gut and seawater samples was extracted using a PowerFecal^TM^ DNA Isolation kit (MO BIO Laboratories, Carlsbad, CA, United States). The 16S rRNA genes were partially amplified using bacterial universal V3–V4 primers 338F and 806R and sequenced using an Illumina MiSeq platform as previously reported (Shi et al., 2019).

PE reads were joined with FLASH using the default settings (Magoč and Salzberg, 2011). The joined pairs were then processed using QIIME v1.9.0 (Caporaso et al., 2010). In brief, the reads were quality filtered at Q20 using the script *split_libraries_fastq.py*. The sequences that passed the quality filtering were checked for chimeras using UCHIME (Edgar et al., 2011). After the chimeras were removed, the remaining sequences were binned into operational taxonomic units (OTUs, 97% nucleotide similarity) based on identification by UCLUST (Edgar, 2010). The most abundant sequence of each OTU was taxonomically assigned in the Greengenes database (release 13.8) using PyNAST (DeSantis et al., 2006a, b). The OTUs that were not affiliated with a bacterial domain were discarded. The OTU table was a 20 × randomly rarefied subset of 27,800 sequences per sample to avoid unequal sequencing depth. The original MiSeq 16S rRNA sequence data generated in this study have been deposited in the Sequence Read Archive of the DDBJ under accession number DRA008473.

To evaluate the overall differences in the bacterial community of the seawater and crab guts, non-metric multidimensional scaling (NMDS) and an analysis of similarity (ANOSIM) were performed based on Bray–Curtis distance metrics (Clarke, 1993). To identify the indicative bacteria that were associated with each group, the indicators at the OTU level were screened (Dufrêne and Legendre, 1997) when the relative abundance of the OTU in any group was greater than 1% and the indicator values (IndVal) were both significant between groups (*p* < 0.05) and greater than 0.4 using the package labdsv in R (Roberts, 2007). The OTU level was selected because this level of characterization provides the finest taxonomical information.

### NMR-Based Metabolomic Analyses of the Tissue Samples

Tissue NMR experiments were performed as described previously (Shi et al., 2019). For all three tissue ^1^H NMR spectra, the residual water signal of δ 4.7–5.2 and methanol signal of δ 3.34–3.38 were discarded. The spectral region of δ 0.8–9.0 was binned with an equal width of 0.004 ppm (2.4 Hz). The integrated areas of all the bins were normalized to the wet weight of the corresponding tissue for each spectrum to compensate for the overall concentration differences.

The normalized NMR data were subjected to the software package SIMCA-P^+^ (12.0, Umetrics, Umeå, Sweden) for multivariate data analysis. Principal component analysis (PCA) was first performed with the data mean-centered to obtain an overview of group clustering and search for possible outliers. Subsequently, orthogonal projection to latent structure discriminant analysis (OPLS-DA) was conducted with unit-variance scaled data. The OPLS-DA models were validated using a fivefold cross-validation method with *R*^2^*X* and *Q*^2^ as the quality parameters (Trygg et al., 2007) and further evaluated using a cross-validation one-way analysis of variance (CV-ANOVA) approach (Eriksson et al., 2008).

To compare the metabolite effects caused by OA exposure, data are presented as metabolite concentrations in the OA groups relative to the control 380 group (C_750_–C_380_)/C_380_ or (C_1500_–C_380_)/C_380_ where C_380_, C_750_, and C_1500_ are the metabolite concentrations in the 380, 750, and 1500 groups, respectively.

### Statistical Analyses

The survival rate was analyzed using a log-rank (Mantel–Cox) test (GraphPad Prism 5, GraphPad Software, Inc., La Jolla, CA, United States). The correlations between all the detected parameters were evaluated by performing a Pearson correlation analysis of the ANOVA results. A correlation was considered significant if *p* < 0.05. A Mantel test was employed in R software to evaluate the association between the digestive and immune enzyme activities; the seawater and gut bacteria; the stress genes, immune genes, and antioxidant genes; and the metabolites of the gut, muscle, and hepatopancreas. Subsequently, a structural equation modeling (SEM) was used to illustrate the interplay of these factors under OA exposure in Amos 22.0 (IBM, Chicago, IL, United States). The data matrix was fitted using SEM based on the maximum-likelihood estimation method. A favorable model fit was confirmed by χ^2^ (Chi-square)/default model test (χ^2^/DF < 2), the root of the mean square residual (RMR < 0.1), a high goodness-of-fit index (GFI > 0.90), and the root-mean-square error of approximation (RMSEA < 0.05).

## Results

### Water Quality After OA Exposure

The *p*CO_2_ and aqueous CO_2_ levels obviously increased with the input of CO_2_ into the water (Figures 1A,B). Simultaneously, the water pH decreased from 8.06 ± 0.03 to 7.89 ± 0.04 (750 μatm) and 7.69 ± 0.04 (1500 μatm) (Figure 1C). Therefore, CO_2_-induced OA was simulated. During the experiment, the water temperature, DO, and salinity were similar across all the groups, with small variations (Figures 1D–F).

### Effects of OA on the Survival Rate and the Growth Performance of the Swimming Crab

**FIGURE 2:**
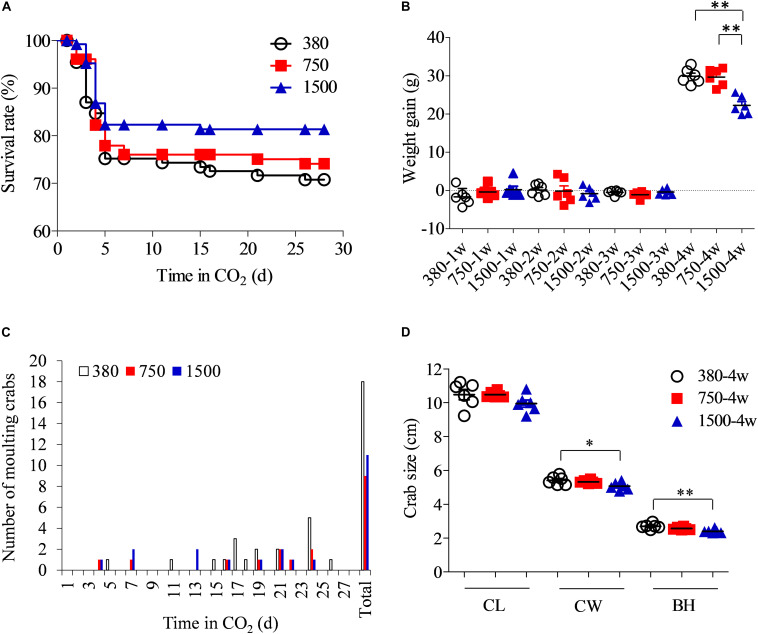
Effects of OA exposure on the survival and growth performance of *Portunus trituberculatus*. **(A)** Survival rate, **(B)** weight gain, **(C)** number of molting crabs, and **(D)** crab size. BH, body height; CL, carapace length; CW, carapace width.

The survival rate of the swimming crabs increased from 75.2% in the 380 group to 76.1% in the 750 group and 82.3% in the 1500 group after the first week of OA treatment (Figure 2A). Mortalities occurred mainly during the first week of OA exposure. After 4 weeks of OA treatment, the survival rate appeared to be higher in crabs from the 1500 group (81.3%), followed by those in the 750 group (74.1%) and the control group (70.8%). At the end of the experiment, the weight gain in the crabs was significantly reduced in the 1500 group compared to that of the other two groups (*p* < 0.05), although there was no significant difference between the 380 and 750 groups (*p* > 0.05, Figure 2B). Weight gain depends mainly on successful molting. More swimming crabs molted in the 380 group than in the two acidified groups (Figure 2C). Concurrently, OA negatively impacted other growth parameters such as CL and BH (Figure 2D).

### Effects of OA on Digestibility and Oxidative and Immunity Stress in the Hepatopancreas

**FIGURE 3:**
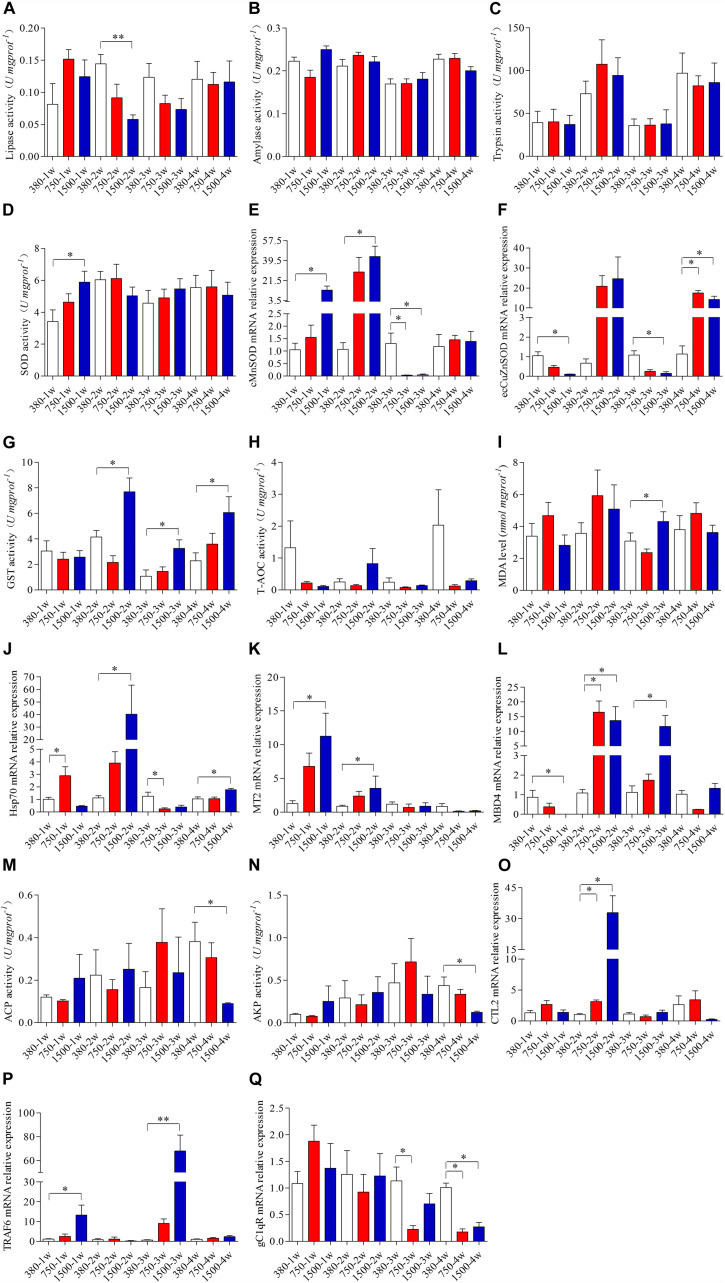
Effects of OA exposure on the digestive enzyme activities, oxidative stress responses, and innate immunity responses of hepatopancreas of *Portunus trituberculatus*. **(A)** Lipase, **(B)** amylase, **(C)** trypsin, **(D)** superoxide dismutase (SOD), **(E)** cMnSOD, **(F)** ecCuZnSOD, **(G)** glutathione-*S*-transferase (GST), **(H)** total antioxidant capacity (T-AOC), **(I)** malondialdehyde (MDA), **(J)** Hsp70, **(K)** MT2, **(L)** MBD4, **(M)** acid phosphatase (ACP), **(N)** alkaline phosphatase (AKP), **(O)** CTL2, **(P)** TRAF6, and **(Q)** gC1qR.

The crabs exposed to 1500 μatm *p*CO_2_ showed significantly decreased activity of lipase after two weeks (*p* < 0.05, Figure 3A). However, no significant effects on the hepatopancreas lipase were found in the crabs exposed to 750 μatm *p*CO_2_ (*p* > 0.05, Figure 3A). No significant effects of elevated *p*CO_2_ were observed for the activities of the hepatopancreas amylase or trypsin (*p* > 0.05, Figures 3B,C).

Environmental stress commonly induces oxidative stress in crustaceans; thus some oxidative stress markers were measured in the hepatopancreas at the biochemical and molecular levels. The 1500 μatm *p*CO_2_ treatment markedly increased the activity of SOD at 1 week (*p* < 0.05, Figure 3D), with a recovery to the control level thereafter. Such a change in enzymatic activity contributed to the significantly altered mRNA expression levels of cMnSOD and ecCuZnSOD (Figures 3E,F). The levels of mRNA expression of cMnSOD significantly increased during the first 2 weeks of 1500 μatm *p*CO_2_ exposure and there was a significant decrease at 3 weeks after exposure to 750 and 1500 μatm *p*CO_2_ exposure (*p* < 0.05, Figure 3E). The mRNA expression levels of ecCuZnSOD significantly decreased at 1 and 3 weeks of 1500 μatm *p*CO_2_ exposure and there was a significant increase at 4 weeks after exposure to 750 and 1500 μatm *p*CO_2_ (*p* < 0.05, Figure 3F). Furthermore, 1500 μatm *p*CO_2_ exposure significantly increased the activity of GST during the last three weeks (*p* < 0.05, Figure 3G). We also observed a marked increase in the MDA level after 3 weeks of 1500 μatm *p*CO_2_ exposure (*p* < 0.05, Figure 3I). However, no significant change in the T-AOC levels was observed after OA exposure (*p* > 0.05, Figure 3H). The expression levels of Hsp70 differed after exposure to 750 and 1500 μatm *p*CO_2_, highlighted by the increased expression at 1 week and reduced expression at 3 weeks in the 750 group as well as an increased expression at 2 and 4 weeks in the 1500 group (*p* < 0.05, Figure 3J). We also noted that 1500 μatm *p*CO_2_ significantly increased the expression of the *MT2* gene during the first 2 weeks (*p* < 0.05, Figure 3K). Furthermore, 750 μatm *p*CO_2_ markedly stimulated the mRNA expression levels of MBD4 at 2 weeks, but 1500 μatm *p*CO_2_ induced a significant change in the mRNA expression levels of MBD4 during the whole period of exposure (*p* < 0.05, Figure 3L).

The immune function is generally impaired when crustaceans are exposed to environmental stress; some immunity indices, therefore, were measured in the hepatopancreas. Our results showed that only the 1500 μatm *p*CO_2_ treatment suppressed the activities of ACP and AKP at 4 weeks (*p* < 0.05, Figures 3M,N). Concurrently, both 750 and 1500 μatm *p*CO_2_ markedly stimulated the mRNA expression level of CTL2 at 2 weeks (*p* < 0.05, Figure 3O). Only 1500 μatm *p*CO_2_ markedly stimulated the mRNA expression level of TRAF6 at 1 and 3 weeks (*p* < 0.05, Figure 3P). In addition, 750 μatm *p*CO_2_ markedly reduced the mRNA expression level of gC1qR during the latter 2 weeks of exposure, whereas 1500 μatm *p*CO_2_ markedly reduced it only at 4 weeks (*p* < 0.05, Figure 3Q).

### Effects of OA on the Seawater and Gut Bacteria

**FIGURE 4:**
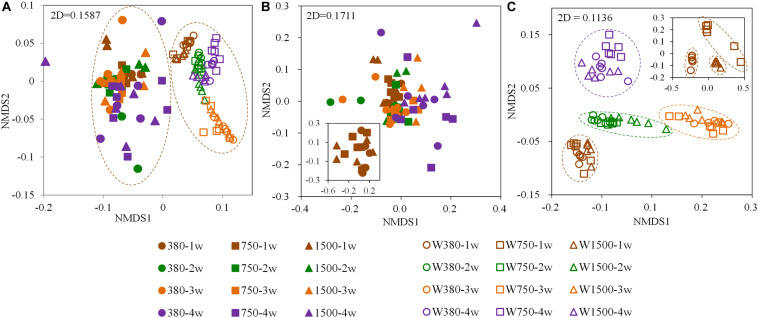
Non-metric multidimensional scaling (NMDS) plots based on the Bray–Curtis distance showing dissimilarities among seawater and *Portunus trituberculatus* gut bacterial communities after OA exposure. **(A)** All bacterial samples, **(B)** gut bacterial samples, and **(C)** seawater bacterial samples.

After quality control, a total of 8,490,028 high-quality sequences were obtained with an average of 51,144 ± 13,106 reads per sample (mean ± SD). Then, the unequal sequencing depths were reduced to 27,800 sequences per sample, resulting in 30,192 OTUs across all samples. The dominant phyla/classes represented in the gut at the 1-week samples included Tenericutes (46.32%), alphaproteobacteria (18.88%), and gammaproteobacteria (10.04%) (Supplementary Figure S1a). However, the dominant phyla/classes found in seawater at the 1-week samples included alphaproteobacteria (64.49%), Bacteroidetes (9.75%), and gammaproteobacteria (6.83%) (Supplementary Figure S1b). Furthermore, the composition of the bacterial community in both the control seawater and control crab gut changed over time. Time appears to have a greater effect than OA exposure on the bacteria (Supplementary Figure S2). However, bacterial community changes related to OA exposure were also observed when compared to the control, despite there being no significant difference in the Shannon index (*p* > 0.05, Supplementary Figure S2). Based on the Bray–Curtis distances of the OTUs detected across samples, an NMDS ordination biplot showed a separation between water and gut bacterial communities (Figure 4A). The NMDS biplot did not exhibit any clear clustering in the gut bacterial communities (Figure 4B) but did show a clear clustering in the aquatic bacterial communities (Figure 4C). The clusters identified from the aquatic bacterial communities were mainly based on developmental time, with subclusters based on *p*CO_2_ concentrations. ANOSIM was used to further corroborate this pattern and revealed a similar bacterial community composition between each of acidified gut tissues and its control counterpart (Supplementary Table S2). However, a significantly distinct bacterial community composition in some acidified seawater samples from their control counterparts was observed (Supplementary Table S3). For example, the bacterial community composition was significantly distinct between the W380 group and the W750 group at 4 weeks.

**FIGURE 5:**
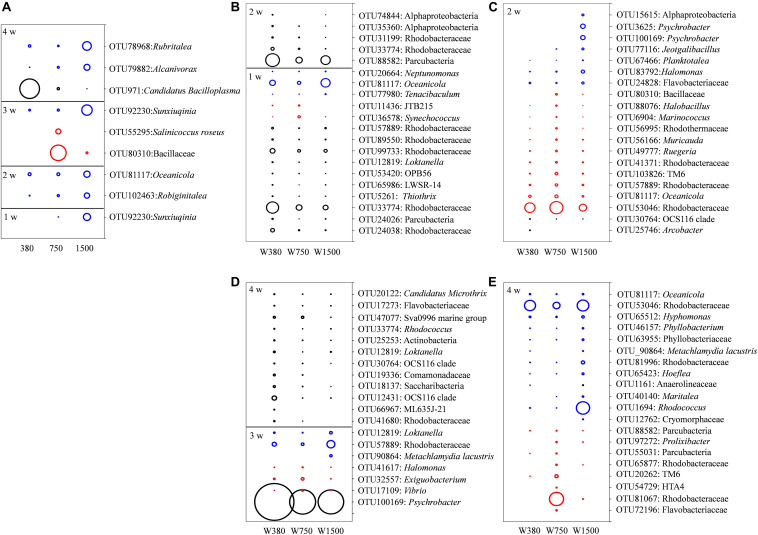
The indicative bacterial OTUs in seawater and crab gut associated with OA exposure. The diameters of the circles are proportional to the relative abundances of the OTUs, with the black, red, and blue circles indicating the peak relative abundances in the seawater and gut samples of 380, 750, and 1500 groups, respectively. The relative abundances of the OTUs in the squares were reduced five times compared with those in the circles. **(A)** The indicative bacterial OTUs in crab gut and **(B–E)** the indicative bacterial OTUs in seawater.

Given the discrete bacterial communities found in the gut and seawater, the assemblages that characterized such differences were studied. Here, the indicators of the dominant (average relative abundance > 1% at least in one group) bacterial OTU level were screened with significant differences between groups (*p* < 0.05) and IndVal values > 0.4. In the gut samples, only one OTU, belonging to *Sunxiuqinia*, was highly relative abundance in the 1500 group at 1 week (Figure 5A). At 2 weeks, two OTUs, belonging to *Robiginitalea* and *Oceanicola*, were the most abundant in the 1500 group. At 3 weeks, two OTUs, belonging to *Salinicoccus roseus* and Bacillaceae, were most abundant in the 750 group, while *Sunxiuqinia* remained the most abundant in the 1500 group. At 4 weeks, *Candidatus bacilloplasma* was the most abundant in the control group, whereas *Alcanivorax* and *Rubritalea* were the most abundant in the 1500 group. In contrast, the water sample presented a substantial change in the composition of the bacterial communities, and many more OTUs were screened during the experiment (Figures 5B–E) (see details in *SI Text, Results: Effects of OA on the seawater bacteria*).

### Effects of OA on the Metabolites of Crab Tissues

**FIGURE 6:**
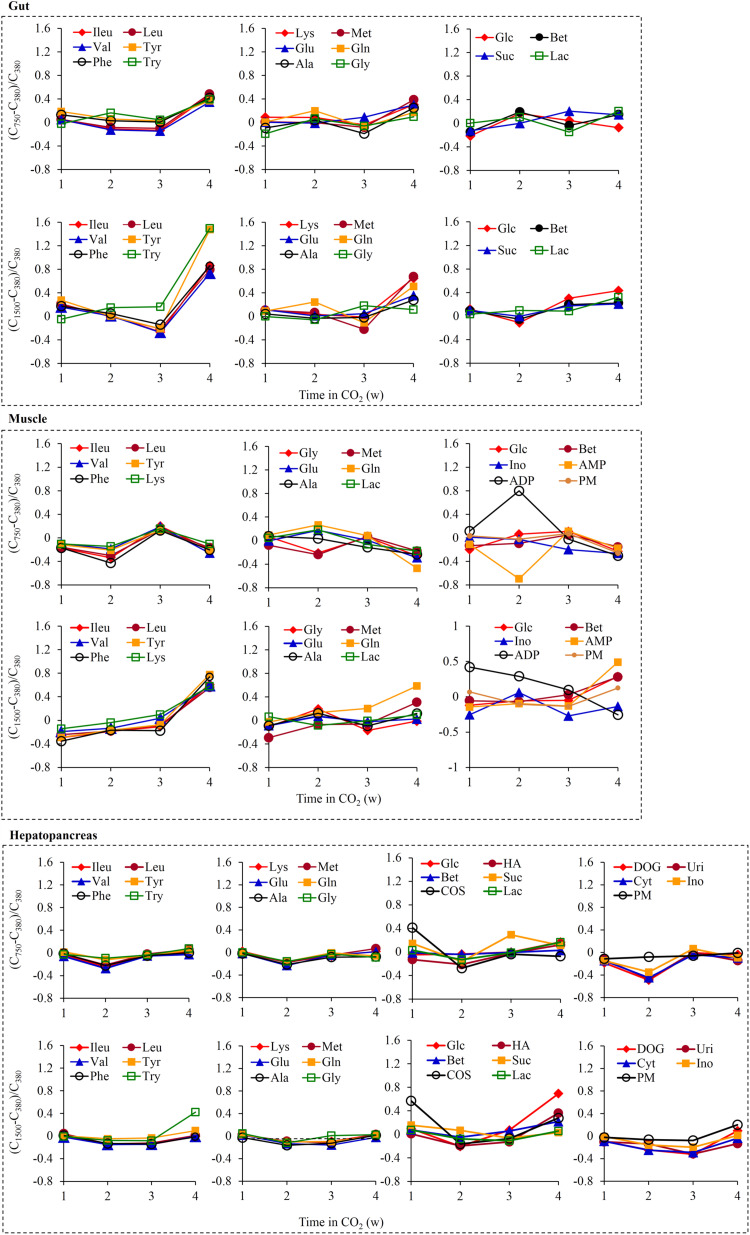
Time dependence of the individual metabolite variations. The values of the *y*-axis were calculated relatively to the levels of the 380 group as (C_750_–C_380_)/C_380_ or (C_1500_–C_380_)/C_380_ where C_750_ and C_1500_ were the respective metabolite concentrations in the 750 group and 1500 group and C_380_ was the metabolite concentration in the 380 group. ADP, adenosine diphosphate; Ala, alanine; AMP, adenosine monophosphate; Bet, betaine; COS, choline-*O*-sulfate; Cyt, cytidine; DOG, deoxyguanosine; Glc, glucose; Gln, glutamine; Glu, glutamate; Gly, glycine; HA, histamine; Ileu, isoleucine; Ino, inosine; Lac, lactate; Leu, leucine; Lys, lysine; Met, methionine; Phe, phenylalanine; PM, 2-pyridinemethanol; Suc, succinate; Try, tryptophan; Tyr, tyrosine; Uri, uridine; Val, valine.

Typical ^1^H NMR spectra from crab gut (Supplementary Figures S3a,b), muscle (Supplementary Figures S3c,d), and hepatopancreas (Supplementary Figures S3e,f) extracts showed a rich metabolite composition (Supplementary Table S4). PCA and OPLS-DA did not show any apparent clustering among the three groups at each sampling time, suggesting a lack of significant differences in metabolic phenotypes in the three tissues between the exposed and control crabs (Supplementary Figure S4). However, some metabolite changes appeared to be associated with OA exposure time (Figure 6). Overall, 1500 μatm *p*CO_2_ caused a stronger effect than 750 μatm *p*CO_2_ on the metabolite changes. Furthermore, the same *p*CO_2_ concentration induced different metabolite changes in three tissues. Exposure to 750 μatm *p*CO_2_ caused a less than 50% increase in the levels of 12 gut amino acids at 4 weeks. Exposure to 1500 μatm *p*CO_2_ caused a higher increase than 750 μatm *p*CO_2_ in the levels of these amino acids, as shown by an ≈ 150% increase in tyrosine and tryptophan levels and a 73–86% increase in the levels of three branched chain amino acids and phenylalanine at 4 weeks. Some changes in the levels of muscle metabolites were observed due to elevated *p*CO_2_. Exposure to 1500 μatm *p*CO_2_ caused a 55–78% increase in the levels of seven amino acids at 4 weeks. It also caused a 42.0% increase at 1 week and a continuous decline thereafter in the ADP level, together with a 49.0% increase in the AMP level at 4 weeks. Furthermore, 750 μatm *p*CO_2_ caused an 80% increase in the muscle ADP level together with a 69.5% decrease in the muscle AMP level at 2 weeks. The hepatopancreas showed the smallest metabolite changes following OA exposure. Exposure to 750 μatm *p*CO_2_ caused a 41.2% increase in the COS level at 1 week as well as an approximately 45% decrease in the deoxyguanosine, uridine, and cytidine levels at 2 weeks. Exposure to 1500 μatm *p*CO_2_ caused a 57.0% increase in the COS level at 1 week as well as a respective 42.3% and 69.4% increase in the tryptophan and glucose levels at 4 weeks.

### Influencing Factors of Crab Survival and Growth

**FIGURE 7:**
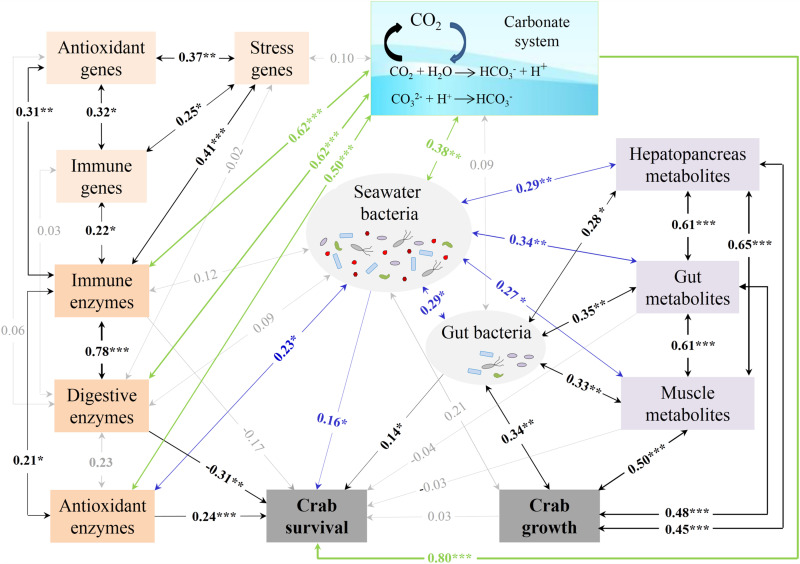
SEM plot for the correlations between the crab survival, growth performance, physiological indices, microbiota, and metabolites. The numbers on the arrows indicate standardized path coefficients, with one-way arrows and two-way arrows indicating unilateral and mutual influences, respectively. The widths of the arrows are proportional to the sizes of numbers. The green, blue, and black lines indicate the significant influence coming from the carbonate system, seawater bacteria, and other factors, respectively. The gray lines indicate no significant influence. *p < 0.05; **p < 0.01; ***p < 0.001.

To further investigate which factor explains growth and survival effects in crabs exposed to elevated *p*CO_2_, correlation analyses between all the variables were performed. Crab growth showed significant (*p* < 0.05 in all cases) positive correlations with the gut and seawater bacteria, three tissue metabolites, and crab survival, whereas crab survival was significantly and positively correlated with the digestive, antioxidant, and immune enzymes; the gut and muscle metabolites; and the gut and seawater bacteria (Supplementary Table S5). Furthermore, the seawater bacteria were positively correlated with the gut bacteria; three tissue metabolites; and the digestive, antioxidant, and immune enzymes. The gut metabolites had positive correlations with the hepatopancreas and muscle metabolites. The digestive enzymes were also positively correlated with the antioxidant and immune enzymes. An SEM model was constructed to further explore the direct/indirect effects of some physiological indices, microbiota, and metabolites on crab growth and survival. The SEM model fit the data well with χ^2^/DF = 0.66, *P* = 0.95, GFI = 0.95, RMSEA = 0.00, RMR = 0.05 and could explain 100% of the variance in both crab growth and survival (Figure 7). The muscle metabolites (λ = 0.50), gut metabolites (λ = 0.48), and hepatopancreas metabolites (λ = 0.45) contributed significantly to the positive direct effects on crab growth. The carbonate system (λ = 0.80), antioxidative enzymes (λ = 0.24), seawater bacteria (λ = 0.16), and gut bacteria (λ = 0.14) contributed significantly to explaining the positive effects on crab survival, and were coupled with a significant negative effect from the digestive enzymes (λ = −0.31). We also observed comprehensive interplay among these indices (see details in *SI Text*, *Results: Interplays among detected indices*), indicating indirect effects on crab growth and survival.

## Discussion

Ocean acidification has comprehensive effects on the growth and development (Long et al., 2013a), physiology and metabolism (Maus et al., 2018), morphology (Ren et al., 2017), and behavior of a variety of marine crabs (Wang et al., 2018). However, the effects of OA have been reported only on the carapace morphology and behavior of juvenile *P. trituberculatus* (Ren et al., 2017, 2018). To the best of our knowledge, this is the first study to reveal the comprehensive effects of OA exposure on *P. trituberculatus*. Our results showed that OA has a mixed effect on swimming crabs, with increased survival and retarded growth. The negative effect on growth is in line with findings published for other crab species such as larvae of *H. araneus* (Walther et al., 2010; Schiffer et al., 2013, 2014; Wang et al., 2018), *Paralithodes camtschaticus*, and *Chionoecetes bairdi* (Long et al., 2013a, b), as well as embryos of *Petrolisthes cinctipes* (Carter et al., 2013; Ceballos-Osuna et al., 2013). Such a consensus between studies performed on different species, at different stages of development and with *p*CO_2_ ranging from 710 to 3100 μatm, strongly supports crab sensitivity to OA. McLean et al. (2018) suggested that the reduction in growth would be due to metabolic suppression, reduced calcification, or energy reallocation. This study provides more information on this question through the analyses of the digestive physiology, antioxidant capacity, stress response, immune function, microbiome, and metabolome.

### Influencing Factors of Retarded Crab Growth

Based on the SEM, three tissue metabolites and gut bacteria appeared to play an important role in crab growth. The elevated *p*CO_2_ elicited an increase in the levels of lactate, glucose, and 12 amino acids in the crab gut, which strongly supports the metabolic depression hypothesis. Metabolic depression may be a beneficial strategy for crabs to respond to an acute stress (Langenbuch and Pörtner, 2004) suggested in other crabs such as *Carcinus maenas* juveniles (Maus et al., 2018) and *P. cinctipes* embryos (Carter et al., 2013), as well as other marine animals (Reipschläger and Pörtner, 1996; Pörtner et al., 1998; Michaelidis et al., 2005; Nakamura et al., 2011) under elevated *p*CO_2_ conditions. OA is capable of enhancing the production of reactive oxygen species (ROS), which has been found to induce oxidative stress in shrimp, *Litopenaeus vannamei* (Wang et al., 2009), and American lobster, *Homarus americanus* (Menu-Courey et al., 2019). A significant increase in the hepatopancreas MDA level further supports the oxidative stress response induced by OA. Oxidative stress inhibited aerobic glycolysis and enhanced protein catabolism (Schock et al., 2010). The increased lactate and glucose levels with elevated *p*CO_2_ are indicative of a change in the energy metabolism from aerobic to anaerobic respiration (Pane and Barry, 2007). The increased lactate level was also observed in dungeness crab (*Cancer magister*) (Pane and Barry, 2007) and in the warm-acclimated spider crab (*Hyas araneus*) (Zittier et al., 2013) subjected to short-term hypercapnia. Increased levels of both glucose and lactate were found in the blue crab (*Callinectes sapidus*) subjected to oxidative stress (Schock et al., 2010). However, our results are in contrast with the results of Hammer et al., who found no change in the glucose level and a significant decrease in the lactate level in the hemolymph of the green shore crab (*Carcinus maenas*) following exposure to elevated CO_2_ (Hammer et al., 2012). One possible explanation for this discrepancy is that very high *p*CO_2_ levels ranging between 2600 and 30,000 μatm were used in experiments on the green shore crab. This transition of respiration mode likely results in an inhibited glucose utilization. In addition, the increased levels of 12 amino acids in the crab gut probably result from protein catabolism. A shift to the catabolism of protein or amino acids occurred in *M. edulis* exposed to elevated CO_2_ levels (Pörtner et al., 1998). Amino acid catabolism was observed in the green shore crabs exposed to elevated CO_2_ (Hammer et al., 2012). Amino acid catabolism serves to produce ammonia to buffer surplus protons induced by OA (Lindinger et al., 1984). However, amino acid catabolism was not measured in the swimming crabs in this study, likely indicating that a new acid–base equilibrium is established in the gut of surviving swimming crabs.

Muscle metabolites were also significantly correlated with crab growth. The elevated *p*CO_2_ induced a transient rise followed by a continuous decline in free ADP levels and a rise in AMP levels, in combination with an increase in free amino acid levels. Decreased ATP/ADP levels were found in the gills of the green shore crab (Hammer et al., 2012) and in the mantles of oysters (*Crassostrea gigas*) (Lannig et al., 2010) exposed to elevated CO_2_. These fluctuations in free ADP and AMP levels suggest an oscillating ATP turnover rate and a disturbed energy homeostasis in the muscle of swimming crabs. This view is supported by findings of the peanut worm (*Sipunculus nudus*) showing oscillations in high-energy phosphate levels during the hypercapnia (Pörtner et al., 1998). Furthermore, the increased AMP levels, which function as a direct agonist of AMP-activated protein kinase (Hardie, 2003), suggest net ATP conservation in the crab muscle at 4 weeks. It is well known that crabs need accumulating HCO_3_^–^ for compensate OA-induced extracellular and intracellular acidosis (Spicer et al., 2007; Tresguerres et al., 2008). Na^+^/K^+^-ATPase is responsible for establishing an ion gradient that can drive carbonic anhydrase, a HCO_3_^–^ exchanger. These two enzymes both require a substantial amount of cellular energy (Pörtner et al., 2011). Thus, cellular energy is reallocated to the acid–base regulation. The gills are regarded as the major organ involved in extracellular ion homeostasis (Henry et al., 2012). The increased energy cost due to ion transport in the gills may elicit energy budget reallocation in other tissues of swimming crabs. Therefore, the energy oscillation in muscle might reflect the energy budget reallocation to reach a new acid–base equilibrium. We also found that 1500 μatm *p*CO_2_ caused an increase in the levels of some amino acids in the muscle at 4 weeks, which may be related to the increased protein catabolism. However, the function of free amino acids in both the gut and muscle remains unknown. It is likely that the function is involved in osmoregulation (Hammer et al., 2012) that is directly linked to acid–base homeostasis (Whiteley et al., 2001). Non-etheless, OA-induced energy oscillation may come at the expense of the growth of swimming crabs.

Hepatopancreas metabolites were also significantly correlated with crab growth. An increase in glucose levels and an overall decrease in amino acid levels in the hepatopancreas provided more supporting information on the metabolism suppression of OA-exposed swimming crabs. These observations indicate amino acid catabolism in the hepatopancreas. We also hypothesized that protein catabolism occurred in this tissue because of a lack of energy needed for the energetically expensive protein synthesis under OA conditions. The enhanced protein and amino acid catabolism likely results in NH_4_^+^ production (Pörtner et al., 1998). Another relevant metabolite is COS, which is produced by choline under hypoxic conditions and by betaine under aerobic conditions. The highly increased COS levels and almost unchanged betaine levels further support acute anoxia in the hepatopancreas under OA exposure. COS can function as an osmoregulatory solute (Hanson et al., 1991; Park and Gander, 1998). However, in this study, it may act to detoxify sulfate by the conjugation of sulfate with choline (Rivoal and Hanson, 1994). This is because elevated *p*CO_2_ leads to the accumulation of sulfate as a counter ion for the elevated cation concentrations under acidified seawater, which has been observed in the hemolymph in Dungeness crabs, *Metacarcinus magister* (Hans et al., 2014). Taken together, the inhibited aerobic glycolysis, energy oscillation, and enhanced protein and amino acid catabolism could partly explain the retarded growth of swimming crabs exposed to elevated *p*CO_2_.

The gut bacteria are usually closely associated with the metabolites of the host (Shi et al., 2019). Thus, it is not surprising to find that the gut bacteria were significantly associated with crab growth. However, only a minor impact was induced by OA exposure on the gut bacteria, suggesting that swimming crabs are capable of keeping the gut bacterial community steady. Non-etheless, OA still caused an overabundance of certain OTUs belonging to *Sunxiuqinia*, *Robiginitalea*, *Oceanicola*, *Alcanivorax*, and *Rubritalea*. These bacteria are residents in the marine environment and some have distinctive ecological functions. For instance, the genus *Alcanivorax* is well known for its ability to degrade *n*-alkanes (Wu et al., 2009; Lai et al., 2016; Konieczna et al., 2018). The genus *Oceanicola* is capable of degrading polycyclic aromatic hydrocarbons (Yuan et al., 2009; Gutierrez et al., 2017). *Robiginitalea* and *Rubritalea* have been characterized as the producers of carotenoids (Song et al., 2018) with antioxidant activities (Shindo and Misawa, 2014). In contrast, OA caused a decrease in the relative abundance of *Candidatus bacilloplasma*. Such a development of bacterial growth and decline in the gut microecological environment indicates a slight disturbance to the healthy gut bacterial community composition. This might trigger the transition of crab fitness from healthy to sub-healthy. This assertion is supported by recent data showing that a dysbiosis in the gut microbial community happens in diseased shrimps and crabs (Xiong, 2018; Shi et al., 2019). The underlying reason for the change in gut microbial community composition is probably reallocation of energy to balance intracellular stress (Hsp70) induced by external pressure (OA pressure in this study), which in turn weakens the ability of the host to filter on alien bacterial species. The energy may be partly reallocated to antioxidant activity (SOD, GST, cMnSOD, and ecCuZnSOD) and immunological activity (ACP, AKP, CTL2, TRAF6, and gC1qR) under OA stress.

### Influencing Factors of Enhanced Crab Survival

The enhanced survival of swimming crabs under OA was expected and observed in this study, despite the lack of enhanced survival in other crabs (Ceballos-Osuna et al., 2013; Long et al., 2013a; Ragagnin et al., 2018; Ramaglia et al., 2018). Based on the SEM, crab survival was mainly explained by the carbonate system, antioxidative enzymes, seawater bacteria, gut bacteria, and digestive enzymes (Figure 7). A substantial body of evidence has shown that OA is a stress to both crustaceans and other marine organisms (Ceballos-Osuna et al., 2013). In this study, the total population of seawater bacterial communities was rapidly and significantly affected by elevated *p*CO_2_, strongly suggesting that seawater bacteria seem to be sensitive to elevated *p*CO_2_ as found in reef biofilms and clam aquaculture water (Witt et al., 2011; Zha et al., 2017). In general, bacteria are flexible and show a potential to adapt to environmental stress. Regarding the short-term (only 4 weeks) exposure in this study, the shifts in the seawater bacterial community probably come from community succession rather than genetic variation. In other words, sensitive bacterial species are replaced by non- or less sensitive ones (Liu et al., 2010). We observed a significant increase in the relative abundance of 22 indicative OTUs, such as *Tenacibaculum* at 1 week and Flavobacteriaceae at 2 weeks, and a significant decrease in the relative abundance of 29 indicative OTUs after OA exposure. These changed OTUs may be the keystone species affected by OA in seawater. However, very few studies are related to the impact of OA exposure on seawater bacteria, except those in biofilms from the Australian Great Barrier Reef and seawater for blood clam farming (Witt et al., 2011; Zha et al., 2017). Nevertheless, a shift in the bacterial community composition means a changed microbial environment for swimming crab, which probably resulted in a changed seawater quality given the important roles played by bacteria in the biogeochemical cycles of marine ecosystems (Liu et al., 2010; Maas et al., 2013). For example, a faster bacterial turnover of polysaccharides at a relatively low ocean pH has been found (Piontek et al., 2010). The global N_2_ fixation potential of *Trichodesmium* could be reduced under acidified conditions (Hong et al., 2017; Luo et al., 2019). Although it is difficult to unravel the exact functions of the bacteria, which are indicative of the seawater status, in this study, their changes did have a significant direct contribution to the survival of swimming crabs. Furthermore, the significance of seawater bacteria may be beneficial not only for crab survival but also for its effects on the gut bacteria, tissue metabolites, and enzyme activity in swimming crabs (Figure 7).

Although the changed gut bacterial community retarded crab growth, it has a significant and beneficial effect on crab survival. One possible reason for this is that bacteria such as *Sunxiuqinia* and *Robiginitalea* are not pathogens. The overabundance of these bacteria can reduce empty niches for pathogen invasion, thus providing a positive contribution to crab survival.

Furthermore, antioxidative enzymes significantly and positively contributed to crab survival. A rapid significant increase in the activities of SOD and GST was observed as well as a quick significant regulation in the mRNA expression of cMnSOD and ecCuZnSOD in the hepatopancreas after OA exposure, indicating a significantly improved antioxidative capacity of swimming crabs. This improved antioxidative capacity may help swimming crabs quickly respond and even eliminate OA-induced oxidative stress, which promotes crab survival.

Digestive enzymes also showed a significant and negative contribution to crab survival; however, among the three digestive enzymes detected, only lipase activity was decreased with the elevated *p*CO_2_ at 2 weeks. The decreased lipase activity likely represents a possible switch from lipid to protein metabolism in the hepatopancreas as previously described in the larvae of the intertidal crab *P. cinctipes* (Carter et al., 2013). This metabolic transition covers the energetic cost in response to elevated *p*CO_2_ and may limit crab survival.

## Conclusion

Ocean acidification led to an antioxidative response, immune responses, metabolic depression, and changed gut bacteria in the swimming crabs via direct generalized oxidative stress and/or an indirect effect of seawater bacteria. These active responses effectively enhanced survival, but at the cost of the growth of swimming crabs. Furthermore, these active responses might endow swimming crabs with a faster response when first facing acidified seawater and improve the transgenerational flexibility of this species. This is because similar features have been found in the low-salinity tolerant swimming crabs (Ye et al., 2014). Importantly, seawater bacteria presented not only a direct contribution to crab survival and growth but also an indirect contribution through the significant interplay with physiological indices, gut bacteria, and tissue metabolites. However, the functions of seawater bacteria are complex and largely unknown and require further study in the future.

## Data Availability Statement

The datasets generated for this study can be found in the DNA Data Bank of Japan, accession number DRA008473.

## Author Contributions

WL conceived and designed the experiments, analyzed the data, wrote the manuscript, prepared the figures and/or tables, and reviewed drafts of the manuscript. ZR performed the experiments, analyzed the data, and reviewed drafts of the manuscript. CM conceived and designed the experiments and reviewed drafts of the manuscript. YY conceived and designed the experiments, analyzed the data, wrote the manuscript, prepared the figures and/or tables, and reviewed drafts of the manuscript. CW conceived and designed the experiments and reviewed drafts of the manuscript. All authors contributed to the article and approved the submitted version.

## Conflict of Interest

The authors declare that the research was conducted in the absence of any commercial or financial relationships that could be construed as a potential conflict of interest.

## Supplementary Material

FIGURE S1 Relative abundances of the dominant bacterial phyla within the crab gut **(a)** and seawater **(b)** groups after OA exposure. The bacterial phylotypes with relative abundance > 1% in at least one group were selected. Proteobacteria were grouped at the class level.

FIGURE S2 Comparisons of crab gut **(a)** and seawater **(b)** bacterial α-diversity after OA exposure.

FIGURE S3 Typical 600 MHz ^1^H NMR spectra of gut, muscle, and hepatopancreas extracts of *Portunus trituberculatus* in the 380 and 1500 groups at 4 weeks. Resonance assignments are given in Supplementary Table S4. The regions of δ 5.2–9.2 in the three tissue spectra were vertically expanded eight times compared with the region of δ 0.8–4.8. Keys: 1, isoleucine; 2, leucine; 3, valine; 4, ethanol; 5, lactate; 6, threonine; 7, alanine; 8, methionine; 9, glutamate; 10, succinate; 11, glutamine; 12, β-alanine; 13, sarcosine; 14, succinimide; 15, aspartate; 16, choline-*O*-sulfate; 17, lysine; 18, phosphorylcholine; 19, arginine; 20, betaine; 21, taurine; 22, glycine; 23, β-glucose; 24, α-glucose; 25, maltose; 26, uracil; 27, cytidine; 28, uridine; 29, fumarate; 30, tyrosine; 31, histamine; 32, phenylalanine; 33, xanthine; 34, deoxyguanosine; 35, hypoxanthine; 36, inosine; 37, tryptophan; 38, adenosine monophosphate; 39, adenosine diphosphate; 40, 2-pyridinemethanol; 41, formate; 42, residual methanol; and 43, α-CH of glucose and amino acids.

FIGURE S4 Principal component analysis plots of ^1^H NMR spectra for the gut, muscle, and hepatopancreas extracts of *Portunus trituberculatus* after 380 (open symbols), 750 (red symbols), 1500 (blue symbols) μatm *p*CO_2_ exposure at 1 (dots), 2 (squares), 3 (triangles), and 4 (diamonds) weeks.

TABLE S1 The primer sequences used in the present study.

TABLE S2 Community dissimilarity test of the bacterial community structure in the crab gut samples based on analysis of similarity using the Bray–Curtis distance.

TABLE S3 Community dissimilarity test of the bacterial community structure in the seawater samples based on an analysis of similarity using the Bray–Curtis distance.

TABLE S4 ^1^H NMR chemical shifts for metabolites from the gut, muscle, and hepatopancreas extracts of *Portunus trituberculatus*.

TABLE S5 Mantel tests demonstrate the Pearson correlations of the crab survival, growth, physiological indices, microbiota, and metabolites.
