# Supplementary material for: Effects of Elevated pCO2 on the Survival and Growth of Portunus trituberculatus
Source: Front Physiol. 2020 Jul 10;11:750. doi: 10.3389/fphys.2020.00750 (PMC7367060; doi:10.3389/fphys.2020.00750)
Supplement: FIGURE S1 — Relative abundances of the dominant bacterial phyla within the crab gut (a) and seawater (b) groups after OA exposure. The bacterial phylotypes with relative abundance > 1% in at least one group were selected. Proteobacteria were grouped at the class level. [file Data_Sheet_1.docx]

**Results:**

***Effects of OA on the seawater bacteria***

At 1 w, a majority (10 of 15) of the OTUs was more abundant in control crabs relative to exposure groups, including five OTUs belonging to Rhodobacteraceae and five OTUs belonging to *Thiothrix*, *Loktanella*, LWSR-14 of Rickettsiales, OPB56 of Chlorobiales, and Parcubacteria. In contrast, the relative abundances of JTB215 belonging to Clostridiales and *Synechococcus* were the highest in the water from the 750 group, whereas *Oceanicola*, *Tenacibaculum*, and *Neptunomonas* were found in the water from the 1500 group (Figure 5b). At 2 w, 11 of 25 OTUs were the most abundant in the 750 group, including three OTUs belonging to Rhodobacteraceae as well as eight OTUs belonging to *Halobacillus*, *Marinococcus*, *Ruegeria*, *Oceanicola*, *Muricauda*, Bacillaceae, Rhodothermaceae, and TM6 (Dependentiae). In contrast, five OTUs, belonging to Arcobacter, Parcubacteria, Alphaproteobacteria Incertae Sedis, OCS116 clade of Rhizobiales, Candidatus Uhrbacteria of Parcubacteria as well as two OTUs belonging to Rhodobacteraceae had enriched relative abundances in control water with five OTUs belonging to *Jeotgalibacillus*, *Halomonas*, *Planktotalea*, Flavobacteriaceae, Alphaproteobacteria Incertae Sedis, and two OTUs, belonging to *Psychrobacter* in the 1500 group (Figure 5c). At 3 w, one OTU, belonging to *Psychrobacter* was most abundant in control crabs. In contrast, the relative abundances of three OTUs, belonging to *Vibrio*, *Exiguobacterium*, and *Halomonas* were the most abundant in water from the 750 group, whereas three OTUs belonging to *Candidatus Metachlamydia*, *Loktanella*, and Rhodobacteraceae had enriched relative abundances in the water from the 380 group (Figure 5d). At 4 w, a total of 30 OTUs were found in three groups. Among them, 11 OTUs were more abundant in control crabs relative to exposure groups, including seven OTUs belonging to *Saccharibacteria*, *Loktanella*, Comamonadaceae, Flavobacteriaceae, Acidimicrobiales, Acidimicrobiales Incertae Sedis, ML635J-21 of Cyanobacteria, two OTUs belonging to Rhodobacteraceae, and two OTUs belonging to OCS116 clade of Rhizobiales. Furthermore, seven OTUs which belongs to *Prolixibacter*, Flavobacteriaceae, Rhodobacteraceae, *Candidatus* Uhrbacteria, TM6 (Dependentiae), Sva0996 marine group of Acidimicrobiales, and Parcubacteria and two OTUs which belongs to HTA4 of Gammaproteobacteria were most abundant in the water of the 750 group. Ten OTUs were the highest in the water from the 1500 group, including *Rhodococcus*, *Maritalea*, *Hoeflea*, *Phyllobacterium*, *Hyphomonas*, *Candidatus Metachlamydia lacustris*, Anaerolineaceae, Rhodobacteraceae, Phyllobacteriaceae, and Cryomorphaceae (Figures 5d-e).

***Interplays among detected indices***

The carbonate system contributed significant positive direct effects on digestive enzymes (λ = 0.62, *p* < 0.05), immune enzymes (λ = 0.62, *p* < 0.05), antioxidative enzymes (λ = 0.50, *p* < 0.05), and seawater bacteria (λ = 0.38, *p* < 0.05). Seawater bacteria had significant positive direct effects on gut bacteria (λ = 0.29, *p* < 0.05), gut metabolites (λ = 0.34, *p* < 0.05), hepatopancreas metabolites (λ = 0.29, *p* < 0.05), muscle metabolites (λ = 0.27, *p* < 0.05), and antioxidative enzymes (λ = 0.23, *p* < 0.05). Furthermore, the gut bacteria had significant positive direct effects on three tissues metabolites (λ ≈ 0.3, *p* < 0.05). Three tissues metabolites had significant positive direct effects on each other (λ ≈ 0.6, *p* < 0.05). Three types of genes had significant positive direct effects on each other (λ = 0.2-0.4, *p* < 0.05). Immune enzymes had significant positive direct effects on digestive enzymes (λ = 0.79, *p* < 0.05), antioxidative enzymes (λ = 0.21, *p* < 0.05), stress genes (λ = 0.41, *p* < 0.05), antioxidative gene (λ = 0.31, *p* < 0.05), and immune genes (λ = 0.22, *p* < 0.05).

**Supporting Information**

The captions of supplementary figures and tables were shown in following. Relative abundances of the dominant bacterial phyla within *Portunus trituberculatus* gut (a) and seawater (b) groups after OA exposure. The bacterial phylotypes with relative abundance > 1 % in at least one group were selected. Proteobacteria were grouped at the class level. Comparisons of *Portunus trituberculatus* gut (a) and seawater (b) bacterial α-diversity after OA exposure. Typical 600 MHz ^1^H NMR spectra of gut, muscle, and hepatopancreas extracts of *Portunus trituberculatus* in the 380 and 1500 groups at 4 w. Principal component analysis plot of ^1^H NMR spectra for the gut, muscle, and hepatopancreas extracts of *Portunus trituberculatus* after 380 (open symbols), 750 (red symbols), 1500 (blue symbols) μatm exposure at 1 (dots), 2 (squares), 3 (triangles), and 4 (diamonds) w. The primers sequences used in the present study. Community dissimilarity test of bacterial community structure in *Portunus trituberculatus* gut samples based on an analysis of similarity using the Bray-Curtis distance. Community dissimilarity test of the bacterial community structure in the seawater samples based on analysis of similarity using the Bray-Curtis distance. ^1^H NMR chemical shifts for metabolites from the gut, muscle, and hepatopancreas extracts of *Portunus trituberculatus*. Mantel tests demonstrate the Pearson correlations of the crab survival, growth, physiological indices, microbiota, and metabolites.

**Figure S1**

**Figure S2**

**Figure S3**

**(c)**

**(e)**

**(d)**

**(f)**

**(a)**

**(b)**

**Figure S4**

**Table S1.** The primers sequences used in this study.

| Genes | Primer sequences (5’-3’) | GenBank number |
| --- | --- | --- |
| TRAF6 | F: TGCGTGTGGTGTTCAATGTA | KP_341006.1 |
|  | R: CTTGTGCCGAGAGAGAGGTG |  |
| CTL2 | F: ACTGGAGGAGAGAAACA | JX_195096.1 |
|  | R: ACTGGAGGAGAGAAACA |  |
| gC1qR | F: ACGTGTCTTCAGGGGCGTGT | MK_076886.1 |
|  | R: CACGAGAGGTGACCGGGAGC |  |
| HSP70 | F: CGTATCCCTAAGACCCAGAAA | FJ_527835.1 |
|  | R: AGAGAGGAGTCACATCCAACA |  |
| MDB4 | F: AAGAGGTTGAAAGGGGAGGG | MH_107034.1 |
|  | R: AGCCAAACAGAAAGAGGAAG |  |
| MT2 | F: GACAAGTGTGTGTGCCAGGAG | KC_203334.1 |
|  | R: CAGCAGGAGCAAGGCTTAGTG |  |
| ecCuZnSOD | F: CGGTAGTGAACTTTGTGCC | FJ_152103.1 |
|  | R: GAAGCCGTGTTGACCTGGA |  |
| cMnSOD | F: ATCCATCACACCAAGCACC | FJ_031018.1 |
|  | R: ACACATCCAAACCCAGCAG |  |
| β-actin | F: CGAAACCTTCAACACTCCCG | FJ_641977.1 |
|  | R: GATAGCGTGAGGAAGGGCATA |  |

**Table S2.** Community dissimilarity test of bacterial community structure in crab gut samples based on analysis of similarity using the Bray-Curtis distance.

| Groups | 380-1w | | 750-1w | | 380-2w | | 750-2w | | 380-3w | | 750-3w | | 380-4w | | 750-4w | |
| --- | --- | --- | --- | --- | --- | --- | --- | --- | --- | --- | --- | --- | --- | --- | --- | --- |
|  | r | *p* | r | *p* | r | *p* | r | *p* | r | *p* | r | *p* | r | *p* | r | *p* |
| 750-1w | 0.220 | 0.053 |  |  |  |  |  |  |  |  |  |  |  |  |  |  |
| 1500-1w | 0.159 | 0.110 | 0.008 | 0.379 |  |  |  |  |  |  |  |  |  |  |  |  |
| 750-2w |  |  |  |  | -0.006 | 0.476 |  |  |  |  |  |  |  |  |  |  |
| 1500-2w |  |  |  |  | 0.019 | 0.394 | -0.035 | 0.502 |  |  |  |  |  |  |  |  |
| 750-3w |  |  |  |  |  |  |  |  | 0.004 | 0.402 |  |  |  |  |  |  |
| 1500-3w |  |  |  |  |  |  |  |  | -0.035 | 0.672 | 0.028 | 0.281 |  |  |  |  |
| 750-4w |  |  |  |  |  |  |  |  |  |  |  |  | -0.120 | 0.953 |  |  |
| 1500-4w |  |  |  |  |  |  |  |  |  |  |  |  | 0.006 | 0.377 | 0.046 | 0.305 |

*p* < 0.05, significant differences between bacterial communities in each group pair.

**Table S3.** Community dissimilarity test of bacterial community structure in seawater samples based on analysis of similarity using the Bray-Curtis distance.

| Groups | W380-1w | | W750-1w | | W380-2w | | W750-2w | | W380-3w | | W750-3w | | W380-4w | | W750-4w | |
| --- | --- | --- | --- | --- | --- | --- | --- | --- | --- | --- | --- | --- | --- | --- | --- | --- |
|  | r | *p* | r | *p* | r | *p* | r | *p* | r | *p* | r | *p* | r | *p* | r | *p* |
| W750-1w | 0.220 | 0.079 |  |  |  |  |  |  |  |  |  |  |  |  |  |  |
| W1500-1w | 0.130 | 0.108 | 0.763 | 0.002 |  |  |  |  |  |  |  |  |  |  |  |  |
| W750-2w |  |  |  |  | 0.406 | 0.016 |  |  |  |  |  |  |  |  |  |  |
| W1500-2w |  |  |  |  | 0.239 | 0.069 | 0.459 | 0.014 |  |  |  |  |  |  |  |  |
| W750-3w |  |  |  |  |  |  |  |  | 0.023 | 0.326 |  |  |  |  |  |  |
| W1500-3w |  |  |  |  |  |  |  |  | 0.322 | 0.030 | 0.026 | 0.301 |  |  |  |  |
| W750-4w |  |  |  |  |  |  |  |  |  |  |  |  | 0.548 | 0.011 |  |  |
| W1500-4w |  |  |  |  |  |  |  |  |  |  |  |  | 0.052 | 0.254 | 0.524 | 0.001 |

*p* < 0.05, significant differences between bacterial communities in each group pair.

**Table S4.** ^1^H and ^13^C NMR data and assignments of the metabolites in gut, muscle, and hepatopancreas extracts of *Portunus trituberculatus*.

| Key | Metabolites | Moieties | δ^1^ H (ppm) and multiplicity^a^ | δ^13^ C (ppm) | Tissue |
| --- | --- | --- | --- | --- | --- |
| 1 | Isoleucine | αCH, βCH, γCH_2_, γ’CH_3_, δCH_3_ | 3.67(d), 1.98(m), 1.26(m), 1.45(m), 1.01(d), 0.94(t) | 62.4, 38.4, 27.3, 17.7, 13.8 | G,M,H |
| 2 | Leucine | αCH, βCH_2_, γCH, δCH_3_, δ’CH_3_ | 3.73, 1.73(m), 1.67(m), 0.98(d), 0.96(d) | 62.4, 42.7, 27.0, 24.7, 23.8 | G,M,H |
| 3 | Valine | αCH, βCH, γCH_3_, γ’CH_3_ | 3.62(d), 2.27(m), 1.04(d), 0.99(d) | 62.9, 31.7, 20.9, 19.6 | G,M,H |
| 4 | Ethanol | αCH_2_, βCH_3_ | 1.19(t), 3.67 | 19.4, 60.5 | M |
| 5 | Lactate | αCH, βCH_3_, COOH | 4.11(q), 1.33(d) | 71.0, 23.0, 185.1 | G,M,H |
| 6 | Threonine | αCH, βCH, γCH_3_ | 3.58(d), 4.26(m), 1.33(d) | 68.7, 63.3, 22.9 | G,M,H |
| 7 | Alanine | αCH, βCH_3_, COOH | 3.79(q), 1.48(d) | 53.4, 19.1, 178.2 | G,M,H |
| 8 | Methionine | αCH, βCH_2_, γCH_2_, S-CH_3_ | 3.85, 2.15(m), 2.65(t), 2.14(s) | 56.9, 32.8, 31.9, 16.9 | G,M,H |
| 9 | Glutamate | δCO, αCH, βCH_2_, γCH_2_, COOH | 3.77(m), 2.12(m), 2.05(m), 2.36(dt) | 184.2, 57.1, 29.8, 36.2, 177.7 | G,M,H |
| 10 | Succinate | CH_2_ | 2.41(s) | 36.8, 184.6 | G |
| 11 | Glutamine | αCH, βCH_2_, γCH_2_, δCO, COOH | 3.77(t), 2.14 (m), 2.46(m) | 57.2, 29.2, 33.8, 180.7, 177.2 | G,M,H |
| 12 | β-alanine | αCH_2_, βCH_2_, COOH | 2.56(t), 3.19(t) | 36.4, 40.5, 181.4 | G |
| 13 | Sarcosine | CH_2_, N-CH_3_, COOH | 3.61(s), 2.74(s) | 53.8, 35.7, 174.2 | G,H |
| 14 | Succinimide | CH_2_ | 2.78(s) | 41.9 | G,H |
| 15 | Aspartate | αCH, βCH_2_, γCOOH | 3.94(dd), 2.82(dd), 2.73(dd) | 56.6, 40.1, 180.8 | G |
| 16 | Choline-O-sulfate | αCH_2_, βCH_2_, N-CH_3_ | 4.33, 3.68, 3.14(s) | 68.9, 55.7 | G,M,H |
| 17 | Lysine | αCH, βCH_2_, γCH_2_, δCH_2_, εCH_2_ | 3.76(t), 1.92(m), 1.48(d), 1.73(m), 3.03(t) | 56.9, 32.6, 24.4, 29.3, 41.9 | G,M,H |
| 18 | Phosphorylcholine | αCH_2_, βCH_2_, N-CH_3_ | 4.21, 3.6, 3.21(s) | 74.9, 57.1 | G |
| 19 | Arginine | εC, αCH, βCH_2_, γCH_2_, δCH_2_ | 3.76(t), 1.92(m), 1.70(m), 3.25(t) | 160.0, 57.3, 31.6, 28.6, 43.4 | G,M,H |
| 20 | Betaine | CH_3_, CH_2_, COOH | 3.27(s), 3.91(s) | 56.2, 69.1, 172.3 | G,M,H |
| 21 | Taurine | CH_2_NH_2_, CH_2_SO_3_ | 3.43(t), 3.27(t) | 38.3, 50.7 | G,M,H |
| 22 | Glycine | αCH_2_, COOH | 3.56(s) | 44.4, 175.6 | G,M,H |
| 23 | β-Glucose | C_1_H, C_2_H, C_3_H | 4.66(d), 3.25(dd), 3.48(m) | 98.9, 77.2, 78.9 | G,M,H |
| 24 | α-Glucose | C_1_H, C_2_H, C_3_H | 5.24(d), 3.54(dd), 3.72(m) | 95.1, 74.9, 75.6 | G,M,H |
| 25 | Maltose | C_4_H, C_7_H, C_8_H | 3.64, 5.4(d), 3.59(d) | 79.6, 102.6, 75.7 | G |
| 26 | Uracil | C_1_, C_2_, C_3_H, C_4_H | 7.54(d), 5.81(d) | 170.2, 156.1, 144.6, 103.4 | G |
| 27 | Cytidine | C_2_, C_4_H, C_5_H, C_6_, C_1’_H, C_2’_H, C_3’_H | 7.85(d), 6.06(d), 5.91(d), 4.33(m), 4.22(t) | 160.5, 144.9, 99.5, 169.1, 93.4, 76.7, 71.9 | H |
| 28 | Uridine | C_2_, C_3_H, C_4_H, C_1’_H | 7.88(d), 5.91(d), 5.91 | 155.5, 144.7, 105.2, 92.1, | G,H |
| 29 | Fumarate | CH, COOH | 6.53(s) | 138.2, 178.0 | G,M |
| 30 | Tyrosine | αCH, βCH_2_, C_1_, Ring C_2,6_H, Ring, C_3,5_H, Ring, C_4_, Ring, COOH | 3.95(dd), 3.20(dd), 3.06(dd), 7.20(d), 6.90(d) | 59.4, 38.4,129.7, 133.6, 118.8, 157.5, 177.1 | G,M,H |
| 31 | Histamine | C_1_H, C_2_H, C_3_, C_4_H_2_, C_5_H_2_ | 8.00(d), 7.06(d), 3.29(t), 3.01(m) | 139.4, 136.1, 122.6, 41.9, 26.9 | G,M |
| 32 | Phenylalanine | αCH, βCH_2_, C_1_, C_2,6_H, C_3,5_H, COOH | 4.00(dd), 3.13(dd), 3.29(dd), 7.33(q), 7.43(t), 7.38(m) | 59.1, 39.4, 137.9, 132.6, 132.4, 131.3,177.1 | G,M,H |
| 33 | Xanthine | C_4_, C_5_, C_6_, C_8_H | 7.91(s) | 152.6, 112.2, 160.5, 144.3 | G |
| 34 | Deoxyguanosine | C_6_, C_5_, C_4_, C_2_H | 8.00(s), 6.30(t) | 144.2, 119.3, 154.0, 140.7 | G,H |
| 35 | Hypoxanthine | C_2_H, C_4_, C_5_, C_6_, C_8_H | 8.19(s), 8.20(s) | 148.6, 121.9, 155.6, 160.5, 145.2 | G,M,H |
| 36 | Inosine | C_1_H, C_2_, C_3_, C_4_H, C_5_, C_1’_H, C_2’_H | 8.35(s), 8.24(s), 6.10(d), 4.77, 4.44(dd), 4.28(dd) | 143.0, 127.2, 161.8, 149.1, 151.5, 91.2, 77.1 | G,M,H |
| 37 | Tryptophan | Ring C_2_H, Ring C_3_, Ring C_4_H, Ring C_5_H, Ring C_6_H, Ring C_7_H, Ring C_8_, Ring C_9_ | 7.34(s), 7.54(d), 7.28 (m), 7.20(m), 7.74(d) | 128.3, 110.0, 115.1, 125.1, 122.3, 121.6, 129.5, 139.5 | G,M,H |
| 38 | Adenosine monophosphate | C_2_H, C_4_, C_8_H, C_1’_H | 8.24(s), 8.58(s), 6.15(d) | 152.5, 151.7, 142.8, 89.6 | M |
| 39 | Adenosine diphosphate | C_2_H, C_4_ ,C_5_ ,C_6_ , C_8_H, C_1’_H | 8.27(s), 8.61(s), 6.14(d) | 155.3, 152.1, 121.8, 158.8, 143.3, 89.7 | M |
| 40 | 2-Pyridinemethanol | C_2_, C_3_H, C_4_H, C_5_H, C_6_H, C_7_H | 8.72(dd), 7.98(td), 8.55(td), 8.04(dd), 4.38(s) | 154.7, 148.4, 130.0, 149.3, 128.8, 49.5 | G,M,H |
| 41 | Formate | CH | 8.46(s) | 174.1 | G |
| 42 | Residual methanol | CH_3_ | 3.36(s) | 51.9 | G,M,H |
| 43 | Glucose and amino acids | αCH resonances | 3.3-3.9 |  | G,M,H |

^a^ Multiplicity: s, singlet; d, doublet; t, triplet; q, quartet; dd, doublet of doublet; dt, doublet of triple; m, multiplet.

^b^ G, gut extracts; M, muscle extracts; H, hepatopancreas extracts.

**Table S5.** Mantel tests demonstrate Pearson correlations of survival, growth, physiological indices, microbiota, and metabolites.

| *p* and r values | Growth | Survival | Digestive enzymes | Antioxidant enzymes | Immune enzymes | Gut metabolites | Hepatopancreas metabolites | Muscle metabolites | Gut bacteria | Water bacteria | Stress genes | Antioxidant genes | Immune genes | Carbonate system |
| --- | --- | --- | --- | --- | --- | --- | --- | --- | --- | --- | --- | --- | --- | --- |
| Growth |  | *0.189* | *-0.061* | *0.052* | *-0.061* | *0.479* | *0.446* | *0.504* | *0.359* | *0.260* | *-0.056* | *-0.080* | *-0.128* | *0.070* |
| Survival | 0.001 |  | *0.152* | *0.589* | *0.152* | *0.106* | *0.046* | *0.130* | *0.2769* | *0.509* | *0.002* | *-0.019* | *0.065* | *0.703* |
| Digestive enzymes | 0.834 | 0.005 |  | *0.230* | *1.000* | *-0.129* | *-0.057* | *-0.083* | *-0.105* | *0.069* | *0.434* | *0.357* | *0.204* | *0.632* |
| Antioxidant enzymes | 0.170 | 0.001 | 0.002 |  | *0.230* | *-0.061* | *-0.096* | *-0.040* | *0.069* | *0.215* | *0.034* | *0.002* | *0.063* | *0.513* |
| Immune enzymes | 0.835 | 0.010 | 0.001 | 0.001 |  | *-0.129* | *-0.057* | *-0.083* | *-0.105* | *0.069* | *0.434* | *0.357* | *0.204* | *0.632* |
| Gut metabolites | 0.001 | 0.004 | 1.000 | 0.926 | 1.000 |  | *0.614* | *0.614* | *0.365* | *0.366* | *-0.098* | *-0.062* | *-0.072* | *-0.015* |
| Hepatopancreas metabolites | 0.001 | 0.119 | 0.790 | 0.996 | 0.825 | 0.001 |  | *0.648* | *0.285* | *0.286* | *-0.023* | *-0.034* | *-0.129* | *-0.027* |
| Muscle metabolites | 0.001 | 0.007 | 0.907 | 0.750 | 0.916 | 0.001 | 0.001 |  | *0.345* | *0.311* | *-0.072* | *-0.071* | *-0.136* | *0.043* |
| Gut bacteria | 0.001 | 0.001 | 0.948 | 0.151 | 0.939 | 0.001 | 0.001 | 0.001 |  | *0.296* | *-0.086* | *-0.001* | *-0.045* | *0.112* |
| Water bacteria | 0.001 | 0.001 | 0.049 | 0.002 | 0.049 | 0.001 | 0.001 | 0.001 | 0.001 |  | *-0.036* | *-0.023* | *0.033* | *0.374* |
| Stress genes | 0.784 | 0.401 | 0.001 | 0.305 | 0.002 | 0.983 | 0.582 | 0.855 | 0.840 | 0.760 |  | *0.379* | *0.247* | *0.125* |
| Antioxidant genes | 0.912 | 0.613 | 0.002 | 0.461 | 0.001 | 0.900 | 0.674 | 0.867 | 0.459 | 0.661 | 0.003 |  | *0.323* | *0.0704* |
| Immune genes | 0.996 | 0.11 | 0.017 | 0.8 | 0.027 | 0.922 | 0.979 | 0.998 | 0.697 | 0.241 | 0.021 | 0.006 |  | *0.006* |
| Carbonate system | 0.081 | 0.001 | 0.001 | 0.001 | 0.001 | 0.663 | 0.665 | 0.184 | 0.029 | 0.001 | 0.048 | 0.124 | 0.426 |  |

*p* value, the numbers in roman (the basic design); r values, the numbers in italics.
